# Genetic and Epigenetic Modulation of Cell Functions by Physical Exercise

**DOI:** 10.3390/genes10121043

**Published:** 2019-12-16

**Authors:** Italia Di Liegro

**Affiliations:** Department of Biomedicine, Neurosciences and Advanced Diagnostics (Dipartimento di Biomedicina, Neuroscienze e Diagnostica avanzata) (Bi.N.D.), University of Palermo, 90128 Palermo, Italy; italia.diliegro@unipa.it; Tel.: +39-091-238-97415/446

**Keywords:** physical activity, physical exercise, aerobic exercise, exercise and health

## Abstract

Since ancient times, the importance of physical activity (PA) and of a wholesome diet for human health has been clearly recognized. However, only recently, it has been acknowledged that PA can reverse at least some of the unwanted effects of a sedentary lifestyle, contributing to the treatment of pathologies such as hypertension and diabetes, to the delay of aging and neurodegeneration, and even to the improvement of immunity and cognitive processes. At the same time, the cellular and molecular bases of these effects are beginning to be uncovered. The original research articles and reviews published in this Special Issue on “Genetic and Epigenetic Modulation of Cell Functions by Physical Exercise” focus on different aspects of the genetics and molecular biology of PA effects on health and, in addition, on the effects of different genotypes on the ability to perform PA. All authors have read and agreed to the published version of the manuscript.

## 1. Introduction

The importance of exercise for health had already been recognized by ancient physicians [1]: for example, Hippocrates (c460–c370 BC) underlined that: “eating alone will not keep a man well, he must also take exercise” [1,2,3]. Moreover, Hippocrates considered exercise like a medicine and was the first recorded physician to provide a detailed written exercise prescription [Tipton, 2014]. Similarly, Herophilus (c335–c280 BC.) believed that exercise and a healthy diet were fundamental for maintaining a healthy body and a healthy mind [4]. Over the centuries, this belief has been expressed and clarified in detail many times [1,5]. Only recently, however, we have been starting to understand the cellular and molecular bases of the detrimental effects on health of a sedentary lifestyle, as well as the reasons why physical activity (PA) can function as a medicine to counteract these effects.

This Special Issue was destined to discuss how cell functions can be modulated at both the genetic and the epigenetic level by physical exercise. Some of the original papers also deal with the effects of gene variants on the ability to perform PA. 

## 2. Effects of Physical Exercise on Cardiovascular Disease, Inflammation, and Immunity

The fact of the matter is that PA can counteract the development of hypertension in a dose-dependent manner [6]. This finding is of high relevance when we consider that hypertension is a widespread disorder as well as the most common risk factor for cardiovascular disease (CVD), which, in turn, is one of the leading causes of death [7,8,9]. In spite of these clear correlations, however, only a few patients with hypertension actually exercise. As discussed in one of the reviews published in this Special Issue, the reasons for the lack of interest in PA of most patients are many; one of them, however, can be found in the genetically determined efficacy of exercise, as shown in a systematic review of studies published in Pubmed and other data banks, describing the association between candidate genes and the antihypertensive effects of exercise [10]. These data have been used to derive a signature of genes related to blood pressure and exercise effects; the corresponding gene exons have been deep-sequenced. From this study, the approach based on deep-sequencing of systematically assembled signature of genes emerges as a cost- and time-efficient way to predict the antihypertensive effects of exercise. The conclusions are relevant for the long-term goal of the described work, that is to develop personalized exercise prescriptions based upon genetic predispositions and other clinical features of the patients [10]. 

A condition that seemingly links PA and CVD is inflammation, evaluated as the number of circulating white blood cells [11]. In their study, published in this Special Issue, Prins and co-workers [12] investigated the association between the genetic substrate and the ability to perform exercise, together with the association between systemic inflammation and PA. Briefly, 68 nucleotide polymorphisms, objectively associated with specific levels of PA, were analyzed, focusing on circulating blood cells. Notably, the authors report that increased PA ability correlates with decreased inflammation and fewer circulating lymphocytes and eosinophils [12].

Modifications induced by acute bouts of exercise were also evidenced in natural killer (NK) cells of the innate immune system, purified from peripheral blood. In particular, in a pilot study described in this Special Issue, Schenk and co-workers found that the DNA methylation level of 25 genes with different regulatory roles was changed in five women after an incremental step test done using a bicycle ergometer [13]. Although this finding is preliminary and new studies are necessary to correlate the methylation of these genes with functional effects, it is anyway of note, since it suggests that exercise can induce epigenetic adaptations, possibly responsible for the improvement of immune system functions.

## 3. Impact of Physical Exercise on Body Mass Index and Lipid Metabolism

Obesity is a leading risk factor for many chronic pathologies, such as CVD and diabetes. The main causes of obesity lie in a positive balance between intake and expenditure of calories, in the consumption of high-energy food [14], and in a sedentary lifestyle, very often also linked to socio-economic conditions [15]. As described in one of the papers published in this Special Issue, a fundamental role for human health and disease is also played by the gut microbial communities (microbiota), initially established at birth but further enriched in the first 3–4 years of life. Importantly, quality and vitality of these communities are influenced by environmental and nutritional cues [16]. 

**Figure 1:**
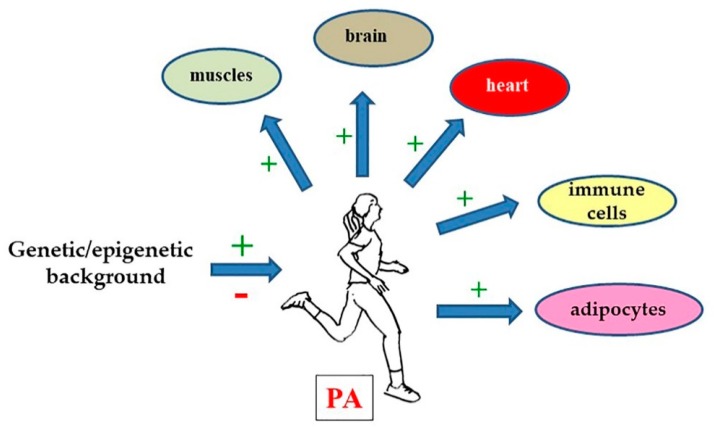
As described in this Special Issue, physical activity (PA) has a beneficial effect on the functions of most tissues in the body, from the heart and circulation to the immune and nervous systems. It can also help in reducing or at least in contrasting obesity, diabetes, and other chronic pathologies, by itself or in association with conventional drugs. However, it is increasingly clear that the genetic (and probably the epigenetic) background of a patient can have both positive and negative effects on the efficacy of exercise. Thus, an important goal for the future should be to identify panels of genes that control the responses to PA.

Obviously, the genetic background also has an impact on obesity, since it influences the susceptibility to metabolic adaptations [17]. Among the genes that have been linked to obesity, that encoding the Fat mass and obesity-associated protein (FTO) is one of the best known [17]. In mice models, it has been shown that some FTO deletions and point mutations cause an increase of metabolic rate, while FTO overexpression induces dyslipidemia [17]. On the other hand, some FTO variants have been associated with obesity in humans, although the underlying molecular mechanisms have not been completely clarified. One interesting observation concerns the fact that FTO is an N6-methyl-adenosine RNA demethylase that might act as a post-transcriptional epigenetic effector [18]. In an original study reported in the present issue, the effect on body mass index (BMI) of the obesity-inducing FTO rs3751812 variant has been analyzed in Taiwanese adults who exercise in comparison with individuals who do not exercise [19]. As expected, individuals with the variant gene both in heterozygosity and homozygosity had a higher BMI than wild-type individuals. However, obesity susceptibility, measured as BMI increase, induced by the FTO variant, was found to be reduced by PA [19]. In another paper of this Special Issue, the authors studied the possible correlation between aerobic exercise and high-density lipoprotein-associated cholesterol (HDL-C) in a large group of 30–70-year-old individuals with different BMI and waist/hip ratio (WHR). The impact of a single nucleotide polymorphism on hepatic lipase (rs1800588 variant) was also analyzed [20]. The results reported in the paper show that, as expected, individuals with abnormal BMI and/or abnormal WHR had significantly lower HDL-C levels with respect to individuals with normal parameters. Notably, aerobic exercise significantly induced higher values of HDL-C in comparison with no exercise [20]. On the other hand, the authors found that in individuals with a CC genotype, HDL-C was not modified by aerobic exercise in any of the BMI and WHR classes. This finding confirms the idea that the effect of PA can be different in different genetic (and perhaps epigenetic) backgrounds (Figure 1).

## 4. Impact of the Genetic Background on PA

As discussed in paragraph 1, the efficacy of exercise can be genetically determined. On the other hand, the ability to perform PA can be influenced by the genotype. In one of the original papers published in this Special Issue, Del Coso and co-workers [21] analyzed the exercise phenotypes of recreational marathon runners endowed with different variants of the gene encoding α-actinin-3 (ACTN3). Normally, the expression of this protein is limited to fast-type muscle fibers. Individuals homozygotes for a null allele (X) of the gene partially compensate by expressing a higher amount of α-actinin-2 in fast-type fibers, a process that, however, does not prevent increased susceptibility to contraction-induced damage [22]. It was reported that the ACTN3 genotype might influence exercise phenotypes in recreational marathoners [22]. In particular, deficiency of α-actinin-3 seems to correlate with higher body fatness, lower muscle strength, and higher muscle flexibility [21].

The role of genetic background in exercise aptitude is also the topic of another original article of this Special Issue. In this work, 451 subjects were genotyped for gene variants correlated with different functional and metabolic parameters (among which inflammation, vascular function, carbohydrate metabolism, and lipid metabolism). Specific questionnaires were also used to evaluate both quantitatively and qualitatively the average physical exercise level [23]. Notably, the authors found that the carriers of a minor allele of the gene encoding glucokinase regulator (GCKR) tended to show a greater frequency of physical exercise in comparison to the major homozygous genotype carriers [23]. 

## 5. Other PA Effects

As a consequence of the hypoxia that often accompanies endurance exercise, oxygen transportation has to be increased in both well-trained and amateur athletes. An increase of oxygen requirement means, in turn, an increase in hemoglobin and myoglobin and thus affects iron concentration and availability, as well as the synthesis of proteins (enzymes, transporters, and regulators) involved in iron and heme metabolism. The levels of mRNAs encoding some of these proteins have been studied by Grzybkowska and co-workers [24] in the leukocytes of 24 amateur runners, before a marathon and 3 h and 24 h after running [24]. The results show a significant modification of gene expression and serum iron and C-reactive protein levels, while ferritin concentration remained unchanged. Notably, the authors found that the running pace also influence the levels of the studied mRNAs [24].

Finally, this Special Issue also includes a review paper that summarizes bibliographic data suggesting that PA improves cognitive processes and memory, together with exerting analgesic and antidepressant effects [25]. The potential mechanisms underlying the effects of PA on brain health are discussed, taking into account hormones, neurotrophines, and neurotransmitters, the release of which is modulated by PA. When already discovered, intra- and extracellular pathways that regulate the expression of some of the genes involved in the neuroprotective and anti-aging effects of PA on brain health are also discussed Notably, experiments are reported that suggest that PA can also function as a medicine in the therapy of neurodegeneration [25].

## 6. Conclusions

In conclusion, this Special Issue has tried to open a forum on the effects of PA on different functions of our body. The results collected concern different aspects of exercise impact, from the effects on the heart and circulation to those on the immune and nervous systems. The results reported in both the reviews and the original papers show that, in most cases, PA can be considered as a real therapy, to be used both alone and in association with conventional drugs. 

Notably, however, in most articles, it is clearly shown that the genetic (and probably the epigenetic) background can affect all the PA effects (Figure 1). Thus, probably, as a long-term goal, the studies on the therapeutic effects of PA should also take into account the specific genome of each athlete or patient considered. Actually, this concept is not new: it is becoming increasingly clear, indeed, that these considerations apply to any therapy, even if we are not yet ready to integrate them into treatment designs.
